# Mortality among acute myocardial infarction patients admitted to hospitals on weekends as compared with weekdays in Taiwan

**DOI:** 10.1038/s41598-022-25415-8

**Published:** 2023-02-09

**Authors:** Sheng-Fu Liu, Chao-Lun Lai, Raymond Nien-Chen Kuo, Ting-Chuan Wang, Ting-Tse Lin, K. Arnold Chan

**Affiliations:** 1 Department of Internal Medicine, National Taiwan University Hospital Hsin-Chu Branch, No.25, Lane 442, Sec. 1, Jingguo Rd., Hsinchu, 30059 Taiwan; 2 Graduate Institute of Clinical Medicine, College of Medicine, National Taiwan University, Taipei, Taiwan; 3 Department of Internal Medicine, College of Medicine, National Taiwan University, Taipei, Taiwan; 4 Institute of Epidemiology and Preventive Medicine, College of Public Health, National Taiwan University, Taipei, Taiwan; 5 Institute of Health Policy and Management, College of Public Health, National Taiwan University, Taipei, Taiwan; 6 Health Data Research Center, National Taiwan University, Taipei, Taiwan; 7 Graduate Institute of Oncology, College of Medicine, National Taiwan University, Taipei, Taiwan

**Keywords:** Cardiology, Health care

## Abstract

Weekend effect has been considered to be associated with poorer quality of care and patient’s survival. For acute myocardial infarction (AMI) patients, the question of whether patients admitted during off-hours have worse outcomes as compared with patients admitted during on-hours is still inconclusive. We conducted this study to explore the weekend effect in AMI patients, using a nationwide insurance database in Taiwan. Using Taiwan National Health Insurance (NHI) claims database, we designed a retrospective cohort study, and extracted 184,769 incident cases of AMI through the NHI claims database between January 2006 and December 2014. We divided the patients into weekend admission group and weekday admission group. Patients were stratified as ST elevation/non-ST elevation AMI and receiving/not receiving percutaneous coronary intervention (PCI). We used a logistic regression model to examine the relative risk of in-hospital mortality and 1-year mortality which were obtained from the Taiwan National Death Registry between study groups. We found no difference between weekend group and weekday group for risk of in-hospital mortality (15.8% vs 16.2%, standardized difference 0.0118) and risk of 1-year mortality (30.2% vs 30.9%, standardized difference 0.0164). There was no statistically significant difference among all the comparisons through the multivariate logistic regression analysis adjusting for all the covariates and stratifying by the subtypes of AMI and whether or not executing PCI during hospitalization. As for AMI patients in Taiwan, admission on weekends or weekdays did not have a significant impact on either in-hospital mortality or 1-year cumulative mortality.

## Introduction

Acute myocardial infarction (AMI) is an important cause of death and ischemic heart disease has consistently ranked among the top ten causes of death in Taiwan^1^. Currently, there are standard practice guidelines for AMI^2–4^. Previous studies have shown that the delayed implementation of specific interventions for patients with AMI can result in worse outcomes^5,6^. Generally, there are fewer employees in the hospital on weekends than on weekdays. Decreased staffing and increased hospital workload are associated with increases in medical adverse events and imply adverse outcomes for ICU patients^7,8^. Many studies have claimed a weekend effect on patient survival rates, i.e. outcomes for patients admitted at the weekends were worse than patients admitted at the weekdays^9,10^. However, for AMI, the question of whether patients admitted during off-hours have worse outcomes as compared with patients admitted during on-hours is still inconclusive^11^. We conducted this retrospective study to explore the weekend effect in patients suffering from AMI, using a nationwide insurance database in Taiwan. This is a subgroup analysis of the previous main research^12^.

## Methods

### Data sources

National Health Insurance (NHI) is mandatory to all citizens in Taiwan. The Taiwan NHI claims database comprises administrative claims data routinely gathered from the NHI operating system. We obtained this research dataset for claims occurring between 2005/1/1 and 2015/12/31 from the NHI claims database. Diagnostic codes were based on the International Classification of Diseases, 9th revision, Clinical modification (ICD-9-CM). We linked our research dataset to the Taiwan National Death Registry and got exact dates of death. In this study, all the personal identifiers were encrypted and all data were analyzed anonymously.

### Study design

This study was a retrospective cohort analysis, of which all patients were adults (≥ 20 years old) having a discharge diagnosis of AMI (ICD-9-CM codes: 410.x) between 2006/1/1 and 2014/12/31. The diagnosis of AMI was further classified as ST elevation myocardial infarction (STEMI) (ICD-9-CM codes: 410.0–410.6 and 410.8), and non-ST elevation myocardial infarction (NSTEMI) (ICD-9-CM codes: 410.7 and 410.9). The index date was defined as the admission date of the index hospitalization for AMI. Weekend admission was defined for patients being admitted to hospital on Saturday, Sunday and national festival days. All other times were defined as weekday admission. Any patient having the same diagnosis within 1 year prior to index date, age younger than 20 years old or admission to psychiatric hospitals would be excluded. For the patient’s referrals between hospitals, only one admission was considered. The outcome measures were in-hospital mortality and 1-year mortality. All patients were followed until death or 365 days after discharge from the index hospitalization whichever came first.

### Background characteristics

Based on Elixhauser’s Comorbidities^13^, underlying comorbidities were defined as appearance of the same diagnostic codes more than twice in outpatient visits or more than once in hospitalizations within 1 year prior to index date. Any medications administered were extracted from the NHI claims database within the 1-year period prior to index date^14^. Only comorbidities and medications with a prevalence of more than 1.0% were retained in the analysis.

We also identified characteristics about the first hospital visited by each patient and age/gender of the attending physician who carried out the percutaneous coronary intervention (PCI) procedures.

### Ethics approval and consent to participate

This study was conducted in accordance with the Declaration of Helsinki. The study protocol was reviewed and exempted from approval by the Institution Review Board of the National Taiwan University Hospital Hsin-Chu Branch. The need of informed consent was waived also by the Institution Review Board of the National Taiwan University Hospital Hsin-Chu Branch.

### Statistical analysis

Continuous variables are presented as means and categorical variables are presented as frequencies. With a sufficiently large sample, a statistical test will almost always demonstrate a significant difference^15^. We used the standardized difference to assess balance of covariates between weekend and weekday groups whereby an absolute standardized difference of greater than 0.10 represented meaningful imbalance^16^. Patients were stratified as STEMI/NSTEMI and receiving/not receiving PCI during the index hospitalization. We applied the logistic regression model to estimate the relative risks (odds ratios [ORs]) of various clinical outcomes to compare weekend group with weekday group by adjustment of background characteristics (all the variables listed in Table 1 and Supplementary Table 1 were included as regressors). We used SAS software, version 9.4 (SAS Institute, Inc., Cary, North Carolina) for data analysis.

**Table 1:** Patient characteristics according to admission time.

	Weekday (N = 130,908)	Weekend (N = 53,861)	Standardized difference	P-value
Age, mean	68.1	67.9	0.0137	0.0018
Male	89,658 (68.5%)	36,748 (68.2%)	0.0056	0.0056
**Comorbidities on admission**				
Congestive heart failure	16,537 (12.6%)	6435 (11.9%)	0.0209	< 0.0001
Cardiac arrhythmias	11,295 (8.6%)	4470 (8.3%)	0.0118	0.0214
Valvular disease	5217 (4.0%)	2144 (4.0%)	0.0002	0.9632
Peripheral vascular disorders	3900 (3.0%)	1510 (2.8%)	0.0105	0.0418
Hypertension, uncomplicated	58,880 (45.0%)	23,779 (44.1%)	0.0167	0.0011
Hypertension, complicated	25,224 (19.3%)	10,131 (18.8%)	0.0117	0.0227
Other neurological disorders	4850 (3.7%)	1846 (3.4%)	0.0150	0.0037
Chronic pulmonary disease	20,039 (15.3%)	7952 (14.8%)	0.0152	0.003
Diabetes, uncomplicated	37,769 (28.9%)	15,271 (28.4%)	0.0110	0.0312
Diabetes, complicated	19,614 (15.0%)	7795 (14.5%)	0.0144	0.005
Renal failure	15,632 (11.9%)	5942 (11.0%)	0.0285	< 0.0001
Liver disease	8006 (6.1%)	3143 (5.8%)	0.0118	0.0214
Peptic ulcer disease excluding bleeding	12,809 (9.8%)	5039 (9.4%)	0.0146	0.0045
Solid tumor without metastasis	7333 (5.6%)	2774 (5.2%)	0.0200	0.0001
Rheumatoid arthritis/collagen vascular diseases	3212 (2.5%)	1304 (2.4%)	0.0021	0.6802
Fluid and electrolyte disorders	3506 (2.7%)	1312 (2.4%)	0.0154	0.003
Blood loss or deficiency anemia	1631 (1.2%)	657 (1.2%)	0.0024	0.6447
Depression	4247 (3.2%)	1744 (3.2%)	0.0004	0.9446
**Medications in past 1 year**				
Antiplatelet	42,337 (32.3%)	16,541 (30.7%)	0.0351	< 0.0001
Anticoagulant	16,089 (12.3%)	5803 (10.8%)	0.0475	< 0.0001
Epilepsy	7905 (6.0%)	3150 (5.8%)	0.0080	0.1172
Hypertension	32,290 (24.7%)	12,853 (23.9%)	0.0187	0.0003
Rheumatic conditions	43,388 (33.1%)	17,460 (32.4%)	0.0155	0.0025
Hyperlipidemia	37,565 (28.7%)	14,996 (27.8%)	0.0190	0.0002
Malignancies	2574 (2.0%)	974 (1.8%)	0.0116	0.0246
Parkinson’s disease	4998 (3.8%)	1943 (3.6%)	0.0111	0.0306
Renal disease	8302 (6.3%)	3043 (5.6%)	0.0292	< 0.0001
End stage renal disease	7648 (5.8%)	2694 (5.0%)	0.0371	< 0.0001
Anti-arrhythmic	16,293 (12.4%)	6320 (11.7%)	0.0218	< 0.0001
Ischemic heart disease/Angina	45,485 (34.7%)	17,240 (32.0%)	0.0581	< 0.0001
Congestive heart failure/Hypertension	72,529 (55.4%)	28,744 (53.4%)	0.0409	< 0.0001
Diabetes	46,992 (35.9%)	18,867 (35.0%)	0.0181	0.0004
Glaucoma	5369 (4.1%)	2046 (3.8%)	0.0155	0.0026
Liver failure	6243 (4.8%)	2397 (4.5%)	0.0152	0.0032
Acid peptic disease	48,949 (37.4%)	19,515 (36.2%)	0.0240	< 0.0001
Respiratory illness/asthma	58,036 (44.3%)	23,555 (43.7%)	0.0121	0.0182
Thyroid disorders	2154 (1.6%)	842 (1.6%)	0.0065	0.2039
Gout	22,904 (17.5%)	9141 (17.0%)	0.0139	0.0068
Pain and inflammation	90,648 (69.2%)	37,104 (68.9%)	0.0077	0.1309
Pain	20,517 (15.7%)	7854 (14.6%)	0.0304	< 0.0001
Depression	15,478 (11.8%)	6257 (11.6%)	0.0064	0.2102
Psychotic illness	19,627 (15.0%)	7711 (14.3%)	0.0191	0.0002
Anxiety and tension	49,383 (37.7%)	19,767 (36.7%)	0.0212	< 0.0001
Ischemic heart disease/Hypertension	82,493 (63.0%)	32,849 (61.0%)	0.0418	< 0.0001
**Subtype of MI on admission**				< 0.0001
STEMI	30,388 (23.2%)	12,566 (23.3%)	− 0.0028	
NSTEMI	33,173 (25.3%)	12,990 (24.1%)	0.0284	
**Percutaneous coronary intervention**				0.0698
Yes	60,911 (46.5%)	24,812 (46.1%)	0.0093	
No	69,997 (53.5%)	29,049 (53.9%)	− 0.0093	
**Clinical outcome**				
In-hospital mortality	21,211 (16.2%)	8494 (15.8%)	0.0118	0.0214
1-year mortality	40,480 (30.9%)	16,248 (30.2%)	0.0164	0.0014

*MI* myocardial infarction, *NSTEMI* non-ST elevation myocardial infarction, *STEMI* ST elevation myocardial infarction.

### Conference presentation

A part of this manuscript (pooled results without stratification by subtypes of acute myocardial infarction and percutaneous coronary intervention or not) had been presented at the European Society of Cardiology (ESC) Congress 2019 held in Paris, France on Sep. 3, 2019.

## Results

### Patient characteristics

There were 184,769 patients identified in the NHI dataset between 2006/1/1 and 2014/12/31. The study population comprised 130,908 admissions on weekdays and 53,861 admissions on weekends. Besides, 42,954 patients were admitted for a STEMI, and 141,815 patients were admitted for a NSTEMI. Table 1 summarizes the patient characteristics across both weekday and weekend groups. There was no significant difference in background characteristics between the weekday and weekend groups. Less than 50% of patients had undergone PCI during hospitalization in both of the groups. The subtypes of myocardial infarction and whether or not executing PCI were not statistically different in both groups (Table 1, Supplementary Table 1).

### Clinical outcomes

The crude in-hospital and 1-year mortality rates of weekday admission were comparable to those of weekend admission. For in-hospital mortality, the weekday group was 16.2% and the weekend group was 15.8% (standardized difference 0.0118). For 1-year mortality, the weekday group was 30.9% and the weekend group was 30.2% (standardized difference 0.0164) (Table 1).

Table 2 shows in-hospital and 1-year mortality stratified by subtypes of myocardial infarction and whether or not executing PCI across both the weekday and weekend groups. The in-hospital and 1-year mortality of both groups were not statistically different within all strata with a standardized difference less than 0.1.

**Table 2:** In-hospital mortality and 1-year mortality according to admission time, stratified by subtype of myocardial infarction and percutaneous coronary intervention.

	Weekday	Weekend	Standardized difference	P-value
**In-hospital mortality**				
STEMI				
PCI	1349/19,162 (7.0%)	554/7815 (7.1%)	− 0.0019	0.8867
No PCI	2331/11,226 (20.8%)	931/4751 (19.6%)	0.0291	0.094
NSTEMI				
PCI	3115/41,749 (7.5%)	1232/16,997 (7.2%)	0.0082	0.3713
No PCI	14,416/58,771 (24.5%)	5777/24,298 (23.8%)	0.0176	0.0213
**1-year mortality**				
STEMI				
PCI	2620/19,162 (13.7%)	1068/7815 (13.7%)	0.0002	0.9881
No PCI	4388/11,226 (39.1%)	1792/4751 (37.7%)	0.0282	0.1042
NSTEMI				
PCI	7093/41,749 (17.0%)	2810/16,997 (16.5%)	0.0122	0.1794
No PCI	26,379/58,771 (44.9%)	10,578/24,298 (43.5%)	0.0272	0.0004

*NSTEMI* non-ST elevation myocardial infarction, *PCI* percutaneous coronary intervention, *STEMI* ST elevation myocardial infarction.

Table 3 shows the results of the multivariate logistic regression analysis stratified by the subtypes of myocardial infarction and whether or not executing PCI after adjusting for all the covariates. Among the STEMI subgroup, there was no difference between the weekend group and weekday group with respect to risk of in-hospital mortality (with PCI: adjusted OR 1.033, 95% confidence interval [CI] 0.929–1.150, p = 0.54; without PCI: adjusted OR 0.934, 95% CI 0.855–1.020, p = 0.13) and risk of 1-year mortality (with PCI: adjusted OR 1.037, 95% CI 0.954–1.128, p = 0.39; without PCI: adjusted OR 0.962, 95% CI 0.891–1.039, p = 0.32). Among the NSTEMI subgroup, there was no difference in risk of in-hospital mortality (with PCI: adjusted OR 0.978, 95% CI 0.912–1.050, p = 0.55; without PCI: adjusted OR 0.979, 95% CI 0.944–1.015, p = 0.26) and risk of 1-year mortality (with PCI: adjusted OR 1.007, 95% CI 0.956–1.061, p = 0.79; without PCI: adjusted OR 0.971, 95% CI 0.940–1.004, p = 0.08) between weekend group and weekday group.

**Table 3:** Relative risk (odds ratio) of in-hospital mortality and 1-year mortality for patients admitted on weekend in comparison to patients admitted on weekday, stratified by the subtype of myocardial infarction and percutaneous coronary intervention.

	OR	95% CI	P-value
**In-hospital mortality**			
STEMI			
PCI	1.033	(0.929, 1.150)	0.54
No PCI	0.934	(0.855, 1.020)	0.13
NSTEMI			
PCI	0.978	(0.912, 1.050)	0.55
No PCI	0.979	(0.944, 1.015)	0.26
**1-year mortality**			
STEMI			
PCI	1.037	(0.954, 1.128)	0.39
No PCI	0.962	(0.891, 1.039)	0.32
NSTEMI			
PCI	1.007	(0.956, 1.061)	0.79
No PCI	0.971	(0.940, 1.004)	0.08

*CI* confidence interval, *NSTEMI* non-ST elevation myocardial infarction, *OR* odds ratio, *PCI* percutaneous coronary intervention, *STEMI* ST elevation myocardial infarction.

## Discussion

In this retrospective cohort analysis, we found no difference in in-hospital and 1-year mortality between the weekday and weekend admissions for AMI in Taiwan. The results remained unchanged after controlling for background characteristics and stratifying the study population into STEMI/NSTEMI and receiving/not receiving PCI.

The weekend effect had been described for higher perinatal mortality of babies born at weekends in 1970s^17^. For major medical emergencies, Bell and Redelmeier reported that there was higher mortality rate among patients admitted on a weekend than patients admitted on a weekday for ruptured abdominal aortic aneurysm, acute epiglottitis and pulmonary embolism but no difference was found for AMI, intracerebral hemorrhage and acute hip fracture^18^. Using the American Acute Coronary Treatment and Intervention Outcomes Network-Get With The Guidelines (ACTION-GWTG) database for analysis, Dasari et al. reported that coronary reperfusion was delayed by an average of 16 min, and the mortality rate was 13% higher in patients presenting off-hours compared with patients presenting on-hours^19^. However, several subsequent studies have reported no significant association between mortality rate and the admission time, in contrast to the study using ACTION-GWTG database^11,20–22^. In Taiwan, the workforce is fewer on weekends than on weekdays indeed. However, cardiologists in Taiwan had dedicated themselves to shortening of door-to-balloon times for patients with STEMI by introducing variable effort and audit programs for many years. Elimination of difference in door-to-balloon times between patients presented on off-hours and on-hours had been achieved^23^. Based on the previous findings, we further disclosed that there was no significant difference in clinic outcomes between AMI patients who were admitted on weekdays and holidays in Taiwan.

The NHI program has been available in Taiwan since 1995, and insures > 99% of 23.4 million residents in Taiwan^24^. The single-payer NHI program in Taiwan provides all insured people comprehensive health care regardless of different social, economic, and health status. Lee et al. found a significant reduction in death rate after the implementation of NHI in Taiwan^25^. Our study showed the prevalence of PCI for patients with AMI was 46%, which was comparable to those in other countries^26^. The findings of our study revealed no difference in short-term and long-term mortality between weekend and weekday admission groups among patients with AMI and this could be attributed to increases in accessibility and the availability of critical care services in Taiwan under the NHI program^27^.

According to previous studies, the mortality rate of STEMI after PCI was about 7%^28^. In our study, the in-hospital mortality rate of STEMI patients in Taiwan after PCI was also about 7% which was comparable to previous data. In the COMPLETE trial, at a median follow-up of 3 years, death from cardiovascular causes or new myocardial infarction occurred in 7.8% of patients in the complete revascularization group while the rate was 10.5% in patients only receiving intervention for culprit lesion^29^. In our study, 1-year mortality among patient with STEMI receiving PCI was about 13% which seemed higher than that reported in the COMPLETE trial. However, the cause of higher long-term mortality in our study was beyond the scope of this paper. It is warranted to conduct a further investigation to explore the possible causes of higher long-term mortality for AMI patients in Taiwan. And we should be more committed to improving the long-term survival rate of STEMI to achieve better long-term outcomes.

### Study limitations

Our study has some limitations. First, we couldn’t extract the exact admission hours from the Taiwan NHI claims database but could only define the weekend admission as admission on Saturday, Sunday and national festival days. Second, our findings only disclosed the current health care status in Taiwan. These findings couldn’t be applied to other healthcare systems in other countries. Third, door-to-balloon time was not available in our NHI claim database. We had no way of knowing whether there was a significance difference in door-to-balloon times between weekend group and weekday group.

## Conclusion

As for AMI patients in Taiwan, admission on weekends or weekdays did not have a significant impact on either in-hospital mortality or 1-year cumulative mortality. The Taiwan NHI system has provided timely accessibility and reliable quality of care for all insured residents suffering from AMI without any difference between admission on weekdays and weekends.

## Supplementary Information

Supplementary Table 1.

## Data Availability

The authors do not own the data underlying this study. The data that support the findings of this study are available from the Health and Welfare Data Science Center (HWDC), Ministry of Health and Welfare, Executive Yuan, Taiwan. The contact information is as follows: Address: No. 488, Sec. 6, Zhongxiao E. Rd., Nangang Dist. Taipei City 11558, Taiwan; Tel: + 886-2-8590-6805; Mr. Young, e-mail address: stsung@mohw.gov.tw. The HWDC must review and approve all applications for use of the database. The process of data analysis has to be conducted in specific offices provided by the HWDC. Only the results of analysis are released after review by the HWDC. The database owned by the HWDC is subject to related regulations of the HWDC including payment, and so is not publicly available.
