# Supplementary material for: Mortality among acute myocardial infarction patients admitted to hospitals on weekends as compared with weekdays in Taiwan
Source: Sci Rep. 2023 Feb 9;13:2320. doi: 10.1038/s41598-022-25415-8 (PMC9911718; doi:10.1038/s41598-022-25415-8)
Supplement: Supplementary file 1 — Supplementary Table 1. [file 41598_2022_25415_MOESM1_ESM.docx]

Supplementary Table 1: Background characteristics about the first hospital visited by each patient and age/sex of the attending physician who carried out the percutaneous coronary intervention procedures

|  | Weekday Group | | | Weekend Group | | |  |  |
| --- | --- | --- | --- | --- | --- | --- | --- | --- |
|  | n=130,908 | | | n=53,861 | | |  |  |
|  | n | % | | n | | % | Standardized difference | P value |
| **Characteristics of hospitals** |  |  | |  | |  |  |  |
| Hospital level |  |  | |  | |  |  | 0.6883 |
| Tertiary center | 54486 | 41.6% | | 22439 | | 41.7% | -0.0008 |  |
| Regional hospital | 36855 | 28.2% | | 15064 | | 28.0% | 0.0041 |  |
| District hospital | 39567 | 30.2% | | 16358 | | 30.4% | -0.0032 |  |
|  |  |  | |  | |  |  |  |
| Teaching hospital or not |  |  | |  | |  |  | 0.7687 |
| Teaching hospital | 117824 | 90.0% | | 48502 | | 90.1% | -0.0015 |  |
| Non-teaching hospital | 13084 | 10.0% | | 5359 | | 9.9% | 0.0015 |  |
|  |  |  | |  | |  |  |  |
| Ownership |  |  | |  | |  |  | < 0.0001 |
| Public hospital | 37474 | 28.6% | | 14722 | | 27.3% | 0.0288 |  |
| Private hospital | 93434 | 71.4% | | 39139 | | 72.7% | -0.0288 |  |
|  |  |  | |  | |  |  |  |
| No. of acute beds |  |  | |  | |  |  | 0.2120 |
| 0~199 | 15042 | 11.5% | | 6343 | | 11.8% | -0.0089 |  |
| 200~399 | 27298 | 20.9% | | 11178 | | 20.8% | 0.0024 |  |
| 400~599 | 24326 | 18.6% | | 10102 | | 18.8% | -0.0044 |  |
| ≥ 600 | 64242 | 49.1% | | 26238 | | 48.7% | 0.0072 |  |
|  |  |  | |  | |  |  |  |
| No. of cardiologists |  |  | |  | |  |  |  |
| Mean (SD) | 10.4 (9.1) | | | 10.2 (9.0) | | | 0.0221 | 0.0003 |
|  |  |  | |  | |  |  |  |
| Volume of cardiac catheterizations one year prior to index date |  |  | |  | |  |  |  |
| Mean (SD) | 1220.0 (1559.6) | | | 1150.5 (1492.0) | | | 0.0455 | < 0.0001 |
|  |  | | |  | | |  |  |
| **Characteristics of physicians** |  | | |  | | |  |  |
| Age of attending physician |  | | |  | | |  |  |
| Mean (SD) | 41.9 (7.9) | | | 41.6 (7.8) | | | 0.0382 | < 0.0001 |
|  |  | | |  | | |  |  |
| Sex of attending physician |  | | |  | | |  | 0.0002 |
| Male | 119570 | | 91.3% | 48927 | 90.8% | | 0.0175 |  |
| Female | 7531 | | 5.8% | 3188 | 5.9% | | -0.0071 |  |
| Unknown | 3807 | | 2.9% | 1746 | 3.2% | | -0.0193 |  |

Abbreviations: SD, standard deviation
